# Selenium and genotypes as marker of risk in BRCA1 mutation carriers

**DOI:** 10.1186/1897-4287-10-S1-A8

**Published:** 2012-01-12

**Authors:** K Jaworska, A Jakubowska, T Huzarski, K Durda, P Serrano-Fernandez, G Sukiennicki, M Muszyńska, T Byrski, J Gronwald, S Gupta, J J Lubiński

**Affiliations:** 1 International Hereditary Cancer Center, Department of Genetics and Pathology, Pomeranian Medical University, Szczecin, Poland

## Aim of the study

Identification of genetic variations in genes related to metabolism of selenium, including glutathione peroxidases (GPX) and thioredoxin reductases (TXNRD) as markers for breast cancer and/or ovarian cancer risks in carriers of BRCA1 gene mutation: analysis of selected changes in the subgroups, depending on the plasma selenium concentration.

## Material and methods

A group of 50 newly diagnosed breast and/or ovarian cancer patients carrying BRCA1 mutation was analyzed by exon-by-exon sequencing of 3 genes (GPX1, GPX4, TXNRD2) coding selenoproteins. Simultaneously a nested case-control study of 39 women with breast cancer and 7 women with ovarian cancer (blood samples for all affected carriers were collected before treatment) and 92 controls matched 1 to 2 cases has been conducted and changes detected by sequencing have been analyzed. Additional case – control studies were performed on 45 pairs 1:2 matched for GPX1 (rs1050450). All cases and controls were matched for age at enrolment, past history of breast cancer and oophorectomy. All these patients were carriers of one of three Polish founder BRCA1 mutation. In these patients plasma selenium level has been determined.

The following techniques for laboratory analyses have been applied: 

a) sequencing on (ABI310), 

b) SimpleProbe or Taqmqan analysis (a melting-curve genotyping with fluorescence-labeled probes based on the LightCycler 480 System (Roche Applied Science)), 

c) determination of selenium concentration in plasma using atomic absorption spectrometer AAnalyst600 (Perkin Elmer).

## Results

The strongest association has been found for GPX1 (rs1050450). Multivariate statistics allowed the analysis of each risk factor separately as well as their interaction with the help of logistic regression model. Subjects having plasma selenium levels under or equal 80 μg/l showed a generally decreased risk of being diseased (OR=0.24; 95%Cl= 0.06-0.94; p=0.041). The genotype of rs1050450 in the gene GPX1 was not a risk factor for itself (p=0.70). However, when analyzed in combination, an interaction effect could be proven where carriers of the minor allele of rs1050450 having plasma selenium levels above or equal 80µg/l were under an increased disease risk (OR=6.24; 95%Cl=1.09-36; p=0.040).

This effect was even more exacerbated among those women which joined the study after adnexectomy (OR=29.2; 95%Cl= 1.6-2000; p=0.0076), while among women without adnexectomy the effect could not be detected at all (OR=2.27; 95%Cl= 0.30-27.8; p=0.43). Adnexectomy was not a risk factor itself in the logistic regression model (p=0.49). Cases and controls in triplets with the same genotypes of GPX1 were also taken into account. Although no significant result was achieved, there was a tendency for more unaffected subjects of CC genotype-carriers in GPX1 with serum selenium levels above or equal 80μg/l (5:14 instead of expected 5:10). The opposite was shown for non-CC genotype carriers in GPX1 with plasma selenium level above or equal 80μg/l (12:18 instead of expected 12:24).

